# PIM-1 contributes to the malignancy of pancreatic cancer and displays diagnostic and prognostic value

**DOI:** 10.1186/s13046-016-0406-z

**Published:** 2016-09-05

**Authors:** Jianwei Xu, Guangbing Xiong, Zhe Cao, Hua Huang, Tianxiao Wang, Lei You, Li Zhou, Lianfang Zheng, Ya Hu, Taiping Zhang, Yupei Zhao

**Affiliations:** 1 Department of General Surgery, Peking Union Medical College Hospital, Chinese Academy of Medical Sciences and Peking Union Medical College, Beijing, 100730 China; 2 Department of General Surgery, Qilu Hospital, Shandong University, Jinan, Shandong Province 250012 China; 3 Key Laboratory of Carcinogenesis and Translational Research (Ministry of Education), Department of Head and Neck Surgery, Peking University Cancer Hospital & Institute, Beijing, 100142 China; 4 Department of Nuclear Medicine, Peking Union Medical College Hospital, Chinese Academy of Medical Sciences and Peking Union Medical College, Beijing, 100730 China; 5 No. 1 Shuaifuyuan, Wangfujing Street, Beijing, 100730 China

**Keywords:** Pancreatic cancer, PIM kinases, Biomarkers, Chemoresistance, Targeted therapy

## Abstract

**Background:**

The effects of PIM-1 on the progression of pancreatic cancer remain unclear, and the prognostic value of PIM-1 levels in tissues is controversial. Additionally, the expression levels and clinical value of PIM-1 in plasma have not been reported.

**Methods:**

The effects of PIM-1 on biological behaviours were analysed. PIM-1 levels in tissues and plasma were detected, and the clinical value was evaluated.

**Results:**

We found that PIM-1 knockdown in pancreatic cancer cells suppressed proliferation, induced cell cycle arrest, enhanced apoptosis, resensitized cells to gemcitabine and erlotinib treatment, and inhibited ABCG2 and EZH2 mRNA expression. Our results indicated that PIM-1 and the EGFR pathway formed a positive feedback loop. We also found that PIM-1 expression in pancreatic cancer tissues was significantly upregulated and that a high level of expression was negatively associated with prognosis (*P* = 0.025, hazard ratio [HR] =2.113, 95 % confidence interval: 1.046–4.266). Additionally, we found that plasma PIM-1 levels in patients with pancreatic cancer were significantly increased and could be used in the diagnosis of pancreatic cancer. High plasma PIM-1 expression was an independent adverse prognostic factor for pancreatic cancer (*P* = 0.037, HR = 1.87, 95 % CI: 1.04–3.35).

**Conclusion:**

Our study suggests that PIM-1 contributes to malignancy and has diagnostic and prognostic value in pancreatic cancer.

**Electronic supplementary material:**

The online version of this article (doi:10.1186/s13046-016-0406-z) contains supplementary material, which is available to authorized users.

## Background

Pancreatic cancer is one of the most deadly malignancies, with an overall 5-year survival rate of 5 % [1]. One of the main reasons for its poor prognosis is that few patients are diagnosed at an early stage [1]; a lack of efficient treatments and resistance to chemotherapy drugs are additional reasons [2]. Despite considerable progress has been made in the understanding of the molecular biology of pancreatic carcinoma [3, 4], the molecular mechanism of pancreatic carcinogenesis remains to be exploited. Investigating the mechanisms of tumour progression, early detection and resensitization of cells to chemotherapy are important for improving the prognosis of patients with pancreatic cancer.

PIM kinases belong to a family of serine/threonine kinases, which is composed of three members (PIM-1, PIM-2 and PIM-3). PIM kinases play pivotal roles in tumour progression and anti-cancer drug resistance [5]. The role of PIM-1 in pancreatic cancer has been investigated. Downregulation of PIM-1 expression was shown to cause cell cycle arrest, increase apoptosis and decrease gemcitabine and intrinsic irradiation resistance in pancreatic cancer cell lines [6, 7]; however, the effects of PIM-1 on cell sensitivity to epidermal growth factor receptor tyrosine kinase inhibitors (EGFR-TKIs) and on cancer stem cells in pancreatic cancer remain unclear. Previous reports have shown that increased EGFR expression has been found in human pancreatic cancer tissue and correlated with a poor prognosis [8, 9]. Epidermal growth factor receptor (EGFR) has also been shown to play key roles in multiple malignant processes involved in cellular proliferation, apoptosis prevention, drug resistance, cancer stem cell marker expression and metastasis in pancreatic cancer [10].

The prognostic value of PIM-1 levels in tissues is controversial. Reiser-Erkan et al. showed that the presence of PIM-1 in pancreatic cancer tissues had a favourable prognostic impact [11]; however, this finding was inconsistent with the oncogenic function of PIM-1 in pancreatic cancer, and further investigations are needed. In addition, the expression levels and clinical value of PIM-1 in plasma have not been reported.

The current study aimed to investigate the roles of PIM-1 in regulating biological behaviours of pancreatic cancer, including proliferation, apoptosis, the cell cycle, gemcitabine and erlotinib sensitivity, and cancer stem cells. We also analysed the expression levels of PIM-1 in tissues, and prognostic values were evaluated. In addition, we measured plasma PIM-1 levels and assessed their potential clinical value for the first time.

## Methods

### Cell culture

Two pancreatic ductal adenocarcinoma (PDAC) cell lines, SW1990 and MiaPaCa-2, were maintained in a humidified incubator with 5 % CO_2_ at 37 °C in RPMI-1640 medium or Dulbecco’s modified Eagle’s medium (DMEM, HyClone, Thermo Fisher Scientific Inc., Waltham, MA, USA) containing 10 % foetal bovine serum (FBS, HyClone).

### siRNA transfection

Human PIM-1 and EGFR siRNAs and control oligos were synthesized by Invitrogen (Shanghai, China). The siRNAs (50–100 nM) were transfected using Lipofectamine 2000 transfection reagent (Invitrogen, Carlsbad, CA, USA) according to the manufacturer’s protocol.

PIM-1 siRNA 5′-GGUGUGUGGAGAUAUUCCUTT-3’

5′-AGGAAUAUCUCCACACACCTT-3’

EGFR siRNA 5′-GGAGAUAAGUGAUGGAGAUTT-3’

5′-AUCUCCAUCACUUAUCUCCTT-3’

Control 5′-UUCUCCGAACGUGUCACGUTT-3’

5′-ACGUGACACGUUCGGAGAATT-3’

### RNA extraction and real-time qRT-PCR

Total RNA was isolated from cells transfected with siRNA or control oligos for 48 h using TRIzol (Invitrogen, Carlsbad, CA) according to the manufacturer’s protocol. Complementary DNA (cDNA) was synthesized from total RNA using a reverse transcription kit (Promega, Madison, WI, USA) according to the manufacturer’s instructions. Real-time qRT-PCR was performed using SYBR Green Master Mix (TaKaRa, Japan) to quantify mRNA levels. GAPDH was used as an internal control. The data were analysed using the 2^-ΔΔCT^ method.

PIM-1 Forward primer GAGAAGGACCGGATTTCCGAC

Reverse primer CAGTCCAGGAGCCTAATGACG

EZH2 Forward primer AATCAGAGTACATGCGACTGAGA

Reverse primer GCTGTATCCTTCGCTGTTTCC

ABCG2 Forward primer CAGGTGGAGGCAAATCTTCGT

Reverse primer ACCCTGTTAATCCGTTCGTTTT

GAPDH Forward primer CGGAGTCAACGGATTTGGTCGTAT

Reverse primer AGCCTTCTCCATGGTGGTGAAGAC

### Growth inhibition assay

The growth inhibition assay was performed using Cell Counting Kit-8 (CCK-8) reagent. To detect the effects of PIM-1 on proliferation, cells were transfected with siRNA or control oligos in 6-well plates for 24 h, trypsinized and reseeded in 96-well plates (1,000 cells/well). CCK-8 (10 μL/well) was added at 0, 24, 48 and 72 h and cultured for 2.5 h at 37 °C. The optical density (OD) was measured using a microplate reader at a wavelength of 450 nm (OD_450_).

To detect the effects of PIM-1 on chemosensitivity, cells transfected with siRNA or control oligos in 6-well plates for 24 h were trypsinized and reseeded in 96-well plates (4,000 cells/well). Then, the cells were incubated with different concentrations of gemcitabine (Gemzar, Eli Lilly and Company, USA) or erlotinib (Tarceva, Roche) for another 48 h. OD_450_ was measured after adding CCK-8 reagent (10 μL/well), and the inhibition rate was calculated.

### Cell cycle assay

Cells transfected in 6-well plates (5 × 10^5^ cells/well) for 48 h were collected, washed with cold phosphate-buffered saline (PBS) and then fixed in 70 % ethanol overnight at 4 °C. After the cells were centrifuged twice (1000 revolutions per minute (rpm) for 5 min each), they were resuspended in 500 μl PBS and then incubated with a solution containing 0.1 % Triton X-100, 10 mg/ml RNase A and 1 mg/ml propidium iodide (PI). Cell cycle analysis was performed using flow cytometry.

### Apoptosis assay

PDAC cells were transfected in 6-well plates. Twenty-four hours later, the cells were treated with gemcitabine or erlotinib. SW1990 cells were treated with 10 μM gemcitabine and 10 μM erlotinib. MiaPaCa-2 cells were treated with 10 μM gemcitabine and 40 μM erlotinib. After the cells were treated for 48 h, they were collected and resuspended in binding buffer. The cells were then stained with annexin V-FITC and PI (Beyotime, China) according to the manufacturer’s instructions and analysed using flow cytometry (FACScan; BD Biosciences, USA).

### Western blot

Total proteins were extracted from cells transfected with siRNA or control oligos for 48 h using RIPA buffer (Applygen, Beijing, China). Total proteins (100 μg) were separated using sodium dodecyl sulphate-polyacrylamide gel electrophoresis (SDS-PAGE) and transferred to polyvinylidene difluoride (PVDF) membranes (Millipore, Billerica, MA, USA). Before the membranes were incubated with primary antibodies overnight at 4 °C, they were blocked with 5 % skim milk at room temperature for 1 h. The membranes were then incubated with horseradish peroxidase–conjugated secondary antibodies at room temperature for 1 h. Bands were visualized using an echochemiluminescence (ECL) detection system. The following primary antibodies were purchased from Cell Signaling Technology: PIM-1 (C93F2) rabbit mAb (3247P), EGF receptor (D38B1) XP® rabbit mAb (4267), phospho-EGF receptor (Tyr1068) (D7A5) XP® rabbit mAb (3777) and β-actin (13E5) rabbit mAb (4970).

### Patient and sample collection

Ninety formalin-fixed, paraffin-embedded pancreatic adenocarcinoma specimens and matched tumour-adjacent tissues were collected and used to construct tissue microarrays, which were then used for detecting PIM-1 protein expression. Ninety preoperative plasma samples were collected from patients with pancreatic cancer and used for detecting plasma PIM-1 levels. None of the patients received neoadjuvant therapy before surgical resection. Preoperative plasma samples from patients with chronic pancreatitis (19), patients with pancreatic neuroendocrine tumours (PNETs, 20), patients with other pancreatic tumours (29) and healthy volunteers (20 cases) were collected as controls. Pancreatic cancer, PNETs and other pancreatic tumours were diagnosed by pathological examination. A diagnosis of chronic pancreatitis was dependent on specific clinical criteria. Blood samples were collected using non-anticoagulant tubes and centrifuged at 3,000 rpm for 10 min. Plasma was collected and stored at −80 °C until use.

### Detection of PIM-1 expression levels using immunohistochemistry (IHC)

Rabbit anti-human PIM-1 polyclonal antibodies (Abgent, AP7932d) were used for staining. Briefly, slides were deparaffinized in xylene and rehydrated in a graded alcohol series. After the slides were washed with PBS, endogenous peroxidase activity was blocked with 3 % H_2_O_2_ for 10 min. Antigen retrieval was carried out by incubating the slides in 0.1 % trypsin and heating them in a microwave oven. Then, the slides were incubated with primary antibody (diluted 1:200) overnight at 4 °C. After the slides were washed three times with PBS, they were incubated with horseradish peroxidase (HRP)-conjugated secondary antibody for 30 min at 37 °C. Diaminobenzidine (DAB) served as a chromogen. The slides were then counterstained with haematoxylin. Nonimmune rabbit serum at the same dilution served as the negative control.

PIM-1 expression levels were assessed according to the intensity of staining and percentage of positive cells. The following scoring system was used for the intensity of stained cells: none (0 points), weak staining (1 point), intermediate staining (2 points) and strong staining (3 points). The following scoring system was given for the percentage of positive cells: absent (0 points), 1–24 % of the cells (1 point), 25–49 % of the cells (2 points), 50–74 % of the cells (3 points), and 75–100 % of the cells (4 points). A final score was calculated by multiplying the above two scores. PIM-1 expression was considered high if the final score was greater than 6 points and low if the final score was 6 points or less.

### Enzyme-linked immunosorbent assay (ELISA)

Plasma PIM-1 levels were measured using a Human PIM-1 ELISA Kit (Catalogue number: CSB-E11825h, CUSABIO, Wuhan, China) according to the manufacturer’s instructions. Carbohydrate antigen 19–9 (CA19-9) levels were detected using a Human CA19-9 ELISA Kit (CSB-E04773h, CUSABIO, Wuhan, China).

### Statistical analysis

Continuous data are displayed as the mean ± standard deviation (SD); these data were compared using analysis of variance (ANOVA), Student’s *t*-test or the Mann-Whitney *U* test. Categorical data are displayed as a percentage; these data were compared using Pearson’s *χ*^2^ test or Fisher’s exact test. A receiver operating characteristic (ROC) curve and the area under the curve (AUC) were used to assess the diagnostic value of plasma PIM-1 levels. The optimal cut-off value, sensitivity and specificity were determined using the Youden Index. AUCs were compared using MedCalc Statistical Software version 13.1.2 (MedCalc Software bvba, Ostend, Belgium; http://www.medcalc.org; 2014). Univariate survival analysis was performed using the Kaplan-Meier method. Cox regression analysis was used for multivariate survival analysis. SPSS v.13.0 software (SPSS, Inc., Chicago, IL, USA) was used for all statistical analyses. *P*-values less than 0.05 were considered statistically significant.

## Results

### Effects of siRNA-mediated knockdown of PIM-1 levels on PDAC cells

**Fig. 1:**
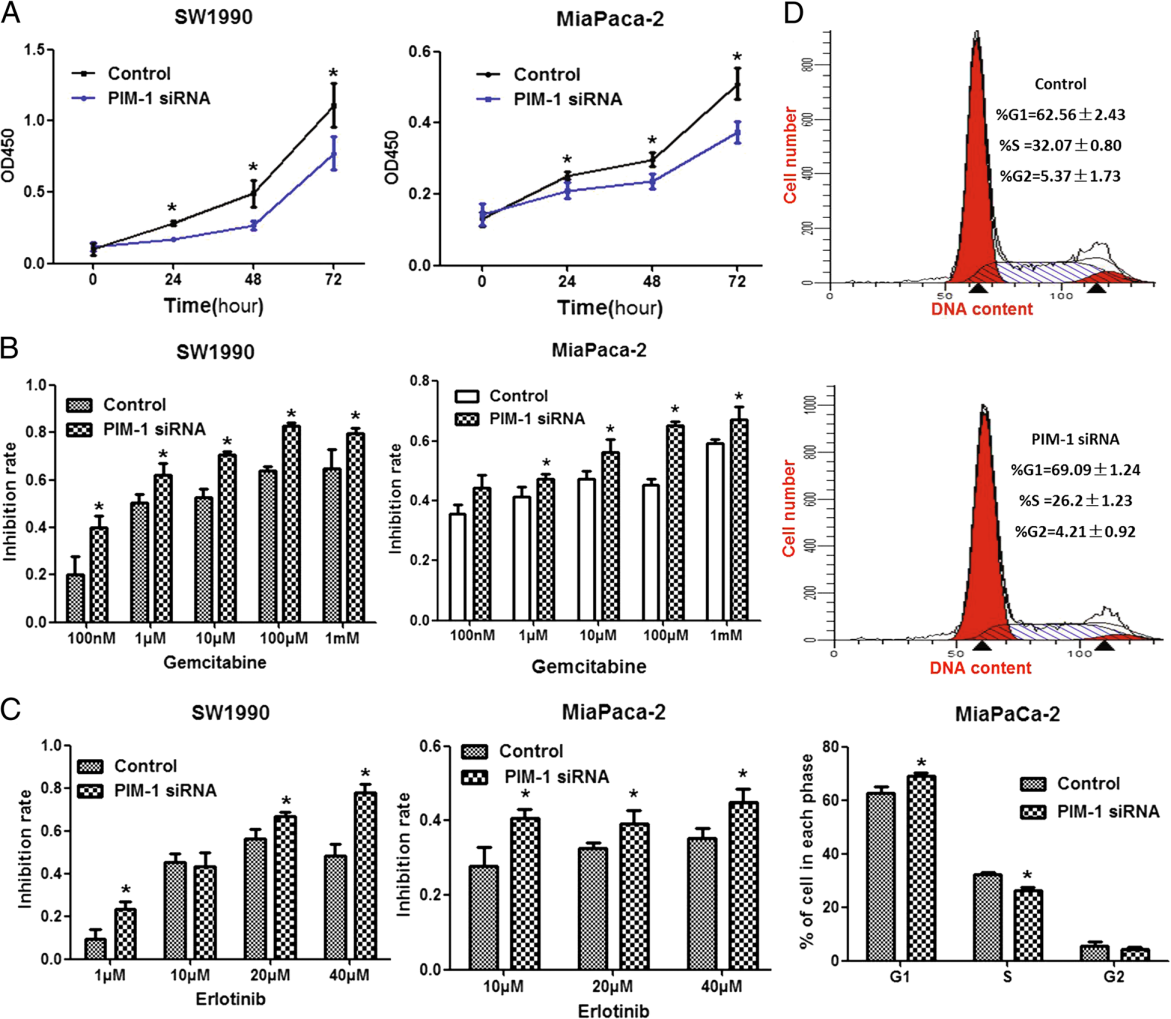
Effects of PIM-1 on proliferation, chemosensitivity and the cell cycle. **a** Knockdown of PIM-1 expression was performed by transfecting PDAC cells with PIM-1 siRNA, and proliferation was detected by CCK8 assay. **b** PDAC cells transfected with PIM-1 siRNA or control oligos were incubated with different concentrations of gemcitabine for 48 h. Cell viability was evaluated by CCK8 assay, and the inhibition rate at each concentration was calculated. **c** Transfected cells were treated with different concentrations of erlotinib; the inhibition rate at each concentration was calculated. **d** MiaPaCa-2 cells transfected with PIM-1 siRNA or control oligos were cultured for 48 h and then collected for cell cycle analysis. The results for the population of cells in each phase of the cell cycle were evaluated by flow cytometry. The data are displayed as the mean ± SD (*, *P* < 0.05)

**Fig. 2:**
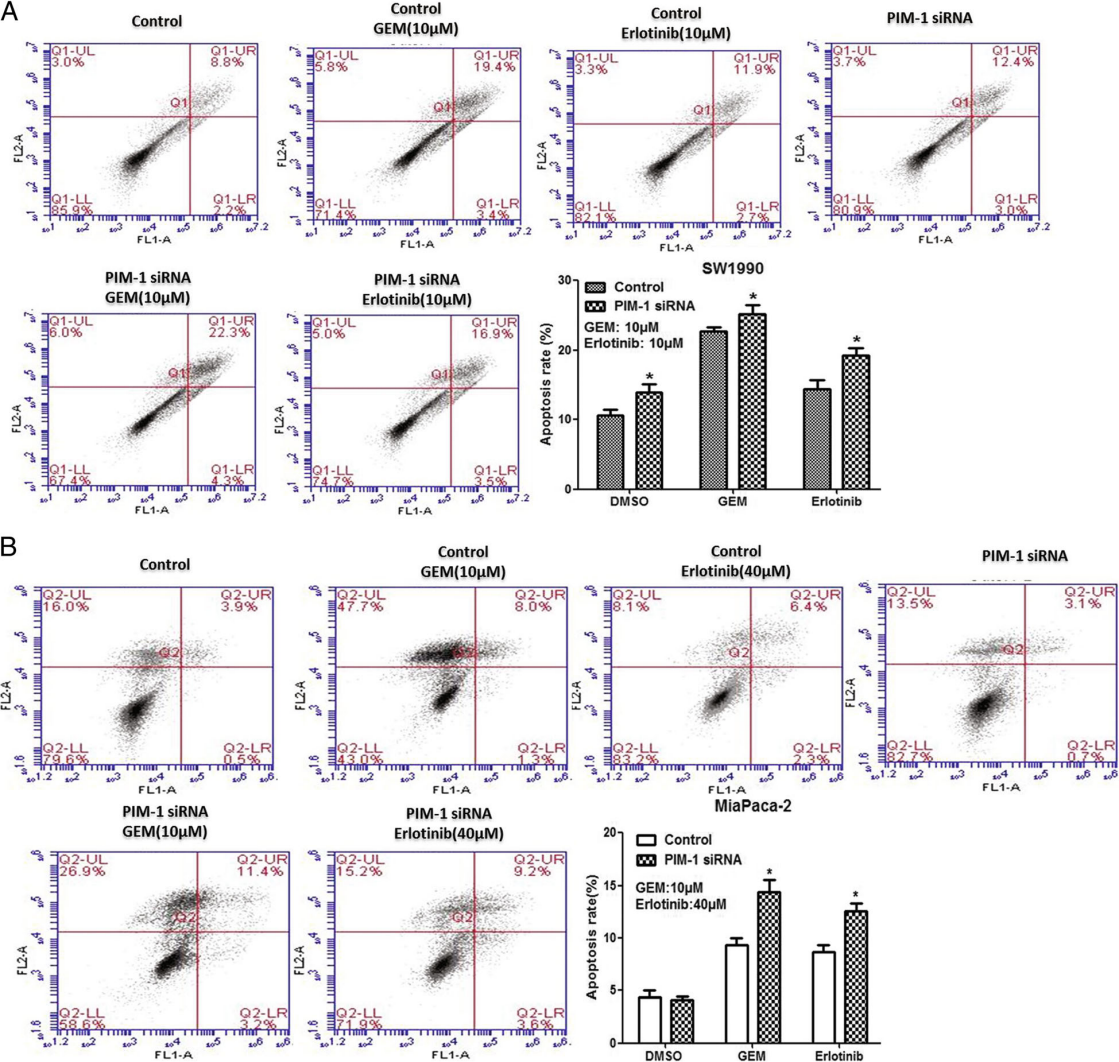
siRNA-mediated knockdown of PIM-1 increased apoptosis of SW1990 and MiaPaCa-2 cells. **a** Flow cytometric analysis of annexin-FITC/PI staining of SW1990 cells that were transfected with PIM-1 siRNA or control oligos and then treated with gemcitabine, erlotinib, or neither for 48 h. **b** Flow cytometric analysis of annexin-FITC/PI staining of transfected MiaPaCa-2 cells that were treated with drugs. The data are displayed as the mean ± SD (*, *P* < 0.05)

We analysed the effect of PIM-1-specific siRNA transfection on PIM-1 expression in PDAC cells by western blot. We found that PIM-1 expression levels in PDAC cells were significantly downregulated after PIM-1 siRNA transfection compared with those of the control group (Additional file 1: Figure S1), which indicated that PIM-1 levels were successfully downregulated by siRNA. CCK8 assay demonstrated that siRNA-mediated knockdown of PIM-1 expression levels in SW1990 and MiaPaCa-2 cells significantly suppressed the proliferation (Fig. 1a) and increased the sensitivity of these cells to gemcitabine and erlotinib treatment (Fig. 1b, c). Flow cytometric analysis showed that downregulation of PIM-1 expression decreased the percentage of S phase cells (Fig. 1d), induced apoptosis (Fig. 2a, b) and promoted gemcitabine- or erlotinib-induced apoptosis (Fig. 2a, b) compared with the control group.

**Fig. 3:**
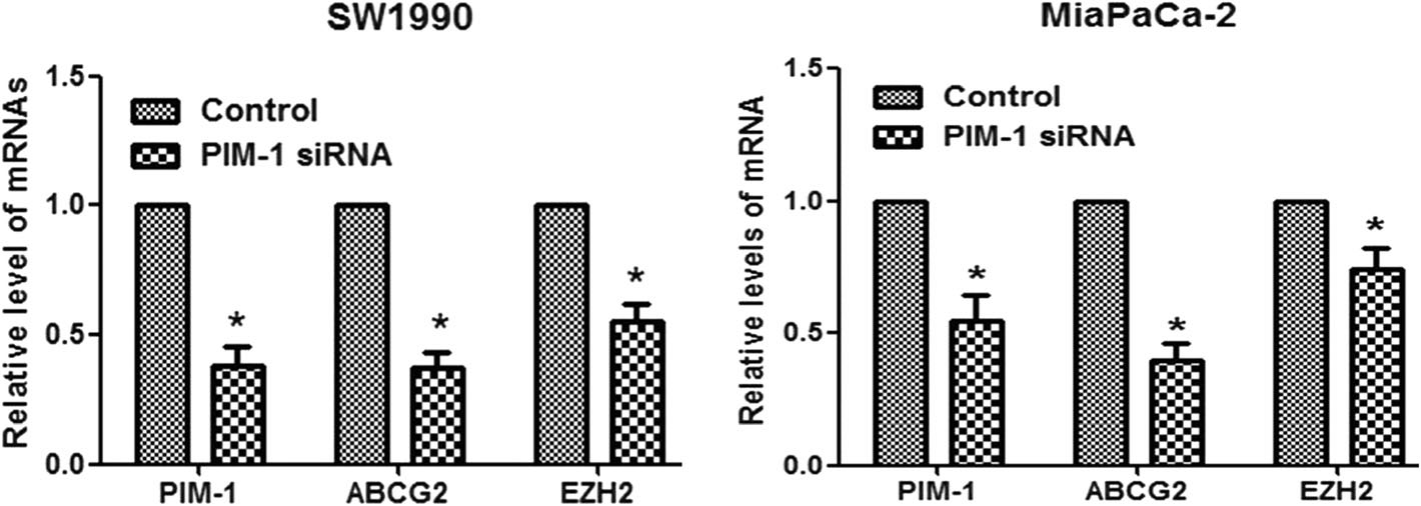
PIM-1 downregulation decreased the expression levels of cancer stem cell markers in pancreatic cancer. qRT-PCR was used to detect the mRNA expression levels of the indicated cancer stem cell markers. GAPDH was used as an internal control. The data are displayed as the mean ± SD (*, *P* < 0.05)

We also investigated the expression levels of the pancreatic cancer stem cell markers ABCG2 [12] and EZH2 [13] by qRT-PCR, and we found that ABCG2 and EZH2 mRNA expression levels were significantly decreased following siRNA-mediated knockdown of PIM-1 expression (Fig. 3).

### PIM-1 and the EGFR signalling pathway form a feedback loop

**Fig. 4:**
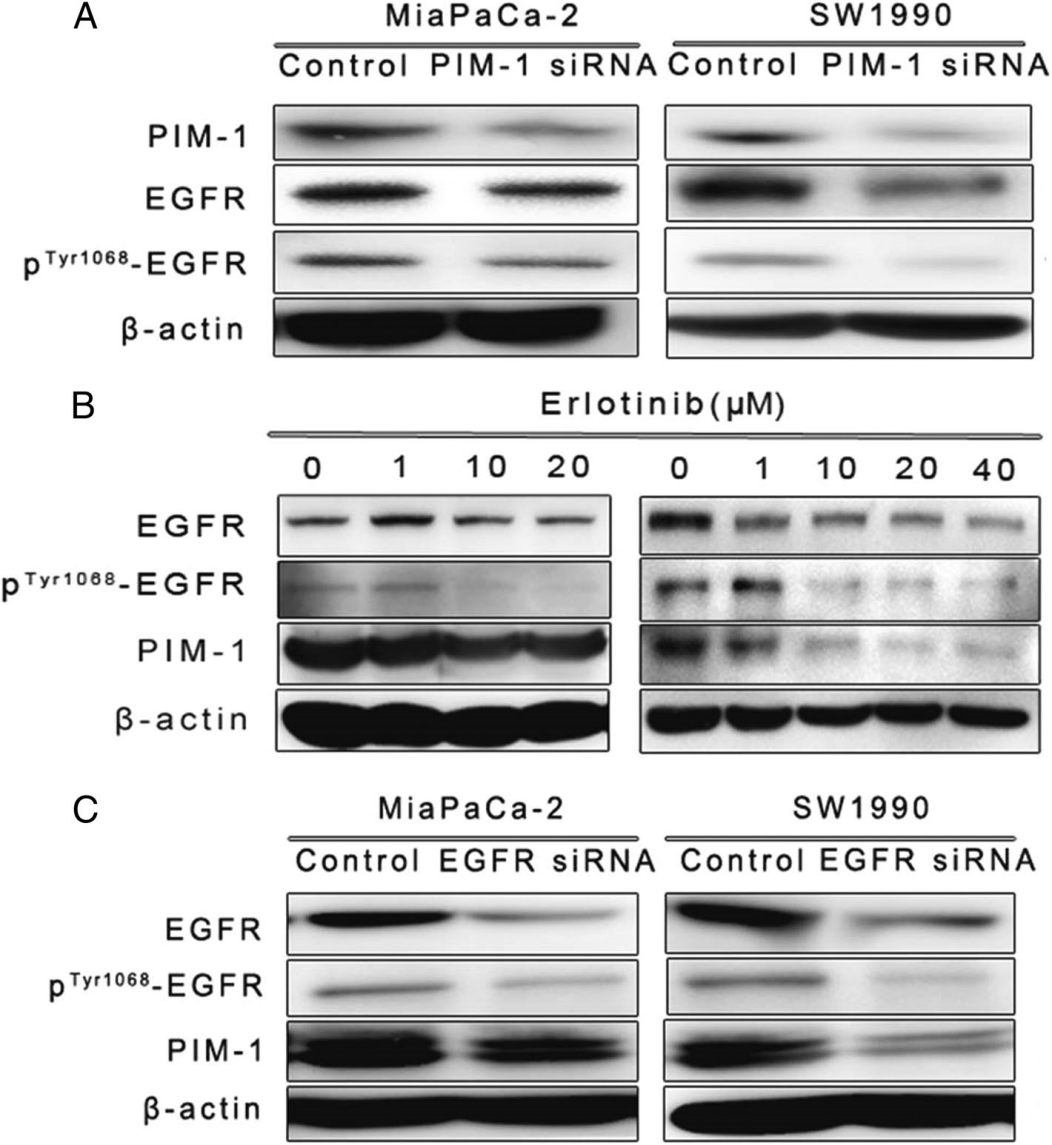
PIM-1 and the EGFR signalling pathway formed a feedback loop. **a** PDAC cells were transfected with PIM-1 siRNA and control oligos for 48 h, and protein levels of PIM-1, EGFR and p^Tyr1068^-EGFR were detected using western blots. **b** PDAC cells were treated with different concentrations of erlotinib to block the EGFR pathway, and protein levels of PIM-1, EGFR and p^Tyr1068^-EGFR were detected using western blots. **c** Knockdown of EGFR expression using siRNA. Western blots were used to detect protein levels of PIM-1, EGFR and p^Tyr1068^-EGFR

We also investigated the mechanisms by which PIM-1 regulates pancreatic cancer progression. We found that PIM-1 and the EGFR signalling pathway formed a feedback loop and that siRNA-mediated knockdown of PIM-1 decreased EGFR and p^Tyr1068^-EGFR expression (Fig. 4a). While blocking the EGFR signalling pathway with erlotinib or EGFR siRNA suppressed PIM-1 expression in SW1990 and MiaPaCa-2 cells (Fig. 4b, c).

### Expression levels and clinical value of PIM-1 in tissues

**Fig. 5:**
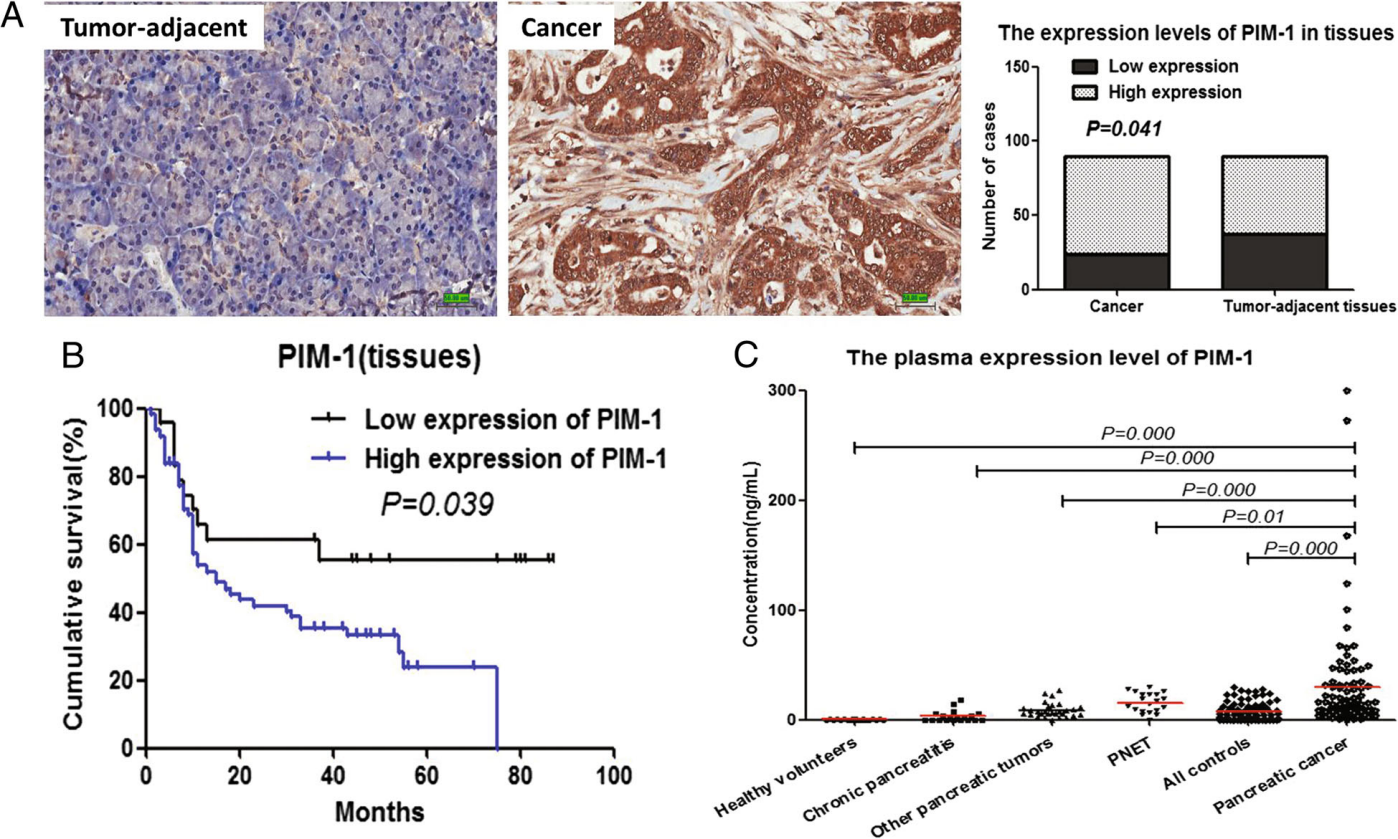
Expression levels and prognostic value of PIM-1 in the tissues and plasma of patients with pancreatic cancer. **a** Expression levels of PIM-1 in tissues were detected using IHC (200× magnification). **b** Kaplan-Meier survival analysis of patients with pancreatic cancer based on PIM-1 expression in tumours. **c** Expression levels of PIM-1 in plasma were detected by ELISA. All controls included healthy volunteers, patients with chronic pancreatitis, patients with other pancreatic tumours and patients with pancreatic neuroendocrine tumours (PNETs)

PIM-1 protein expression levels in tissues were detected using IHC. PIM-1 protein was located in the cytoplasm or in both the cytoplasm and nucleus. Twenty-four of 90 pancreatic cancer samples showed low-level PIM-1 protein expression, and 66 of 90 pancreatic cancer samples showed high-level PIM-1 protein expression. In contrast, 37 of 90 tumour-adjacent samples showed low-level PIM-1 protein expression, and 53 of 90 tumour-adjacent samples showed high-level PIM-1 protein expression. Thus, PIM-1 protein levels were increased significantly in cancer tissues compared with tumour-adjacent tissues (*P* = 0.041) (Fig. 5a).

In addition, we evaluated the clinical value of PIM-1 expression levels in tissues. Eighty-seven of 90 cases with complete medical records were included in the analysis. There was no correlation between the expression levels of PIM-1 and clinicopathological parameters, including sex, age, tumour location, differential degree, TNM staging, diabetes or perineural invasion (Additional file 2: Table S1). Univariate analysis indicated that TNM staging and PIM-1 expression level were potential prognostic factors for pancreatic cancer (Additional file 3: Table S2; Fig. 5b). Multivariate analysis indicated that TNM staging (II/III/IV) and PIM-1 expression (high) were independent adverse prognostic factors (*P* = 0.023, HR = 1.882, 95 % CI: 1.085–3.266; *P* = 0.025, HR = 2.113, 95 % CI: 1.046–4.266, respectively) (Additional file 3: Table S2).

### Expression levels and diagnostic value of plasma PIM-1 levels

Plasma PIM-1 levels in patients with pancreatic cancer have not been described. In the current study, we measured plasma PIM-1 levels and then assessed their diagnostic value in pancreatic cancer. Plasma PIM-1 levels in patients with pancreatic cancer (29.8 ± 47.7 ng/ml) were significantly higher than in healthy volunteers (0.21 ± 0.31 ng/ml), in patients with chronic pancreatitis (3.11 ± 5.2 ng/ml), in patients with other pancreatic tumours (8.75 ± 6.6 ng/ml) or in patients with PNETs (15.7 ± 8.9 ng/ml) (*P* = 0.000, *P* = 0.000, *P* = 0.000 and *P* = 0.01, respectively). In addition, plasma levels of PIM-1 in patients with pancreatic cancer were significantly higher than were those in all control subjects combined (29.8 ± 47.7 ng/ml vs. 7.21 ± 8.3 ng/ml, *P* = 0.000) (Fig. 5c).

**Fig. 6:**
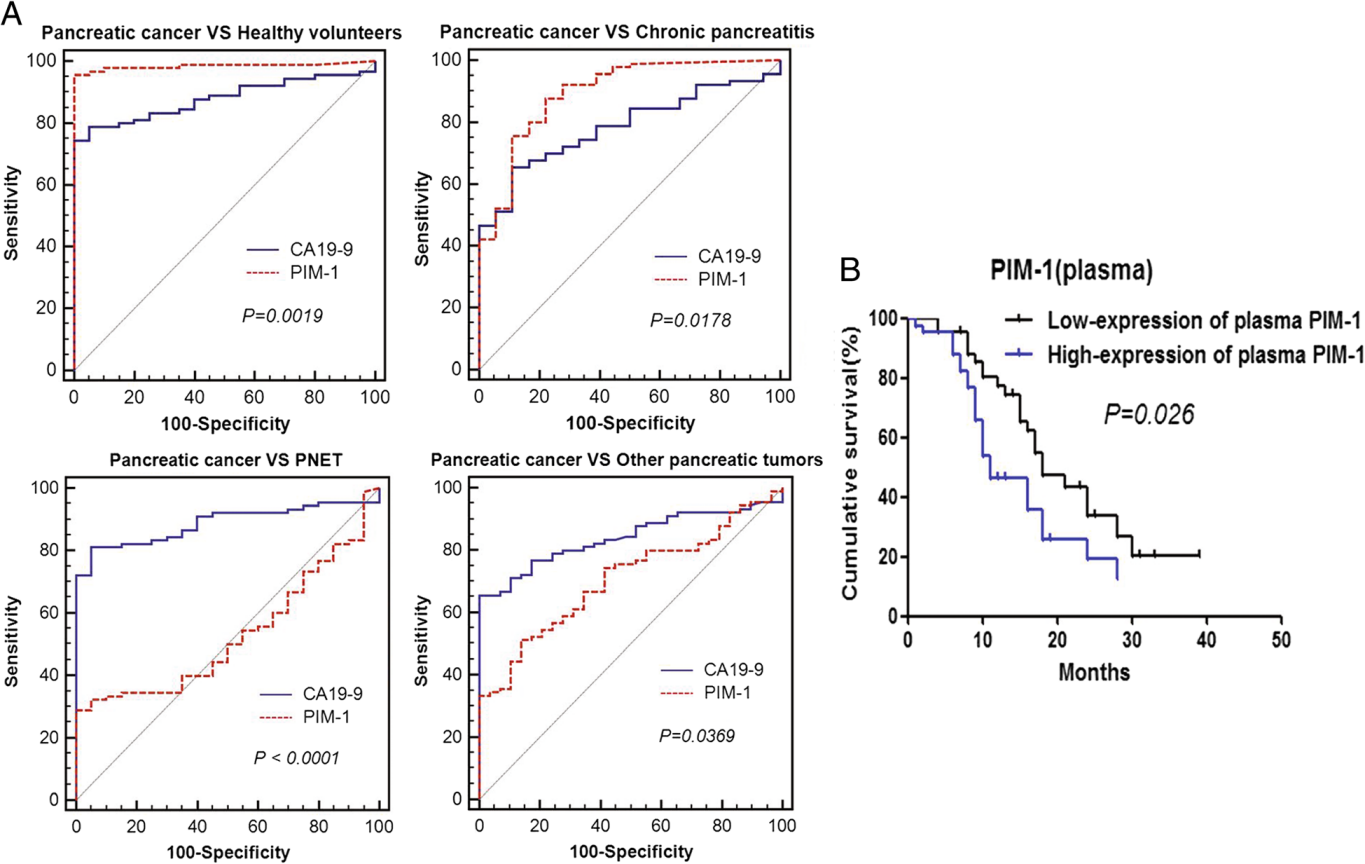
Diagnostic and prognostic value of plasma PIM-1 levels. **a** Diagnostic value of PIM-1 and CA19-9 was analysed using ROC curves. The AUC values were compared using the *Z* test. **b** Kaplan-Meier survival analysis of patients with pancreatic cancer based on PIM-1 expression levels in plasma.

Furthermore, we assessed the diagnostic value of plasma PIM-1 levels using ROC curve analysis. Plasma PIM-1 levels displayed diagnostic values for distinguishing patients with pancreatic cancer from healthy volunteers, patients with chronic pancreatitis, and patients with other pancreatic tumours (*P* = 0.000, *P* = 0.000, and *P* = 0.001, respectively). When patients with pancreatic cancer were distinguished from healthy volunteers, plasma PIM-1 levels were significantly superior to CA19-9 levels (0.984 vs. 0.897, respectively; *P* = 0.0019), particularly in terms of sensitivity (95.6 vs. 74.4 %) (Fig. 6a). When patients with pancreatic cancer were distinguished from patients with chronic pancreatitis, plasma PIM-1 levels were also significantly superior to CA19-9 levels (0.895 vs. 0.785, respectively; *P* = 0.0178), but the specificity was lower (77.8 vs. 88.9 %) (Fig. 6a). The AUC, sensitivity and specificity are shown in Table 1.

**Table 1:** Diagnostic values of plasma PIM-1 and CA19-9 levels

	PIM-1			CA19-9		
	AUC	Sensitivity	Specificity	AUC	Sensitivity	Specificity
		(%)	(%)		(%)	(%)
Pancreatic cancer VS Healthy volunteers	0.984	95.6	100	0.879	74.4	100
Pancreatic cancer VS Chronic pancreatitis	0.895	87.8	77.8	0.785	65.6	88.9
Pancreatic cancer VS Other pancreatic tumors	0.706	51.1	86.2	0.841	65.6	100
Pancreatic cancer VS PNET	0.529	——	——	0.888	81.1	95

Note: *AUC* area under the curve, *CI* confidence interval, *PNET* pancreatic neuroendocrine tumors

### Association between plasma PIM-1 levels and clinicopathologic parameters and survival analysis in patients with pancreatic cancer

Eighty-two patients with pancreatic cancer with complete medical records and follow-up information were divided into high- and low-level expression groups according to median plasma PIM-1 levels. We found that plasma PIM-1 levels were associated with age, tumour location and TNM staging (*P* = 0.031, *P* = 0.000 and *P* = 0.013, respectively) (Additional file 4: Table S3). Patients with high plasma PIM-1 levels had advanced-stage tumours. Univariate analysis showed that M staging, TNM staging, margin status and PIM-1 levels were potential prognostic factors for pancreatic cancer (*P* = 0.000, *P* = 0.000, *P* = 0.002 and *P* = 0.026, respectively) (Additional file 5: Table S4). Multivariate analysis indicated that TNM staging (advanced) and plasma PIM-1 levels (high) were independent adverse prognostic factors (*P* = 0.000, HR = 1.88, 95 % CI: 1.17–2.57; *P* = 0.0037, HR = 1.87, 95 % CI: 1.04–3.35, respectively) (Additional file 5: Table S4, Fig. 6b).

## Discussion

PIM kinases play pivotal roles in the development and progression of pancreatic cancer. PIM-1 is involved in regulating cell proliferation, the cell cycle, apoptosis and chemoresistance in multiple tumours, including pancreatic cancer [5, 14]. PIM-3 is overexpressed in patients with pancreatic cancer and is a prognostic indicator related to poor survival in these patients [15, 16]. Downregulation of PIM-3 expression can decrease cell proliferation, invasion, chemoresistance, radioresistance and angiogenesis in pancreatic cancer [7, 17, 18]. The potential mechanisms by which PIM-3 promotes tumours include upregulation of pSTAT3Try705, pSurvivinThr34 and VEGF, activation of the AKT/β-catenin pathway, phosphorylation of Bad, and inhibition of Bcl-xl [15, 16, 18, 19]. However, the regulatory roles and mechanisms of PIM-1 in pancreatic cancer are still unclear. We found that siRNA-mediated knockdown of PIM-1 inhibited cell proliferation, increased apoptosis, decreased the percentage of S-phase cells, resensitized cells to gemcitabine treatment, and promoted gemcitabine-induced apoptosis, consistent with the results reported in previous studies [6, 7, 20, 21]. We also demonstrated that downregulation of PIM-1 increased the sensitivity of PDAC cells to erlotinib, enhanced erlotinib-induced apoptosis, and decreased cancer stem cell marker expression. To our knowledge, the effects of PIM-1 on erlotinib sensitivity and cancer stem cells in pancreatic cancer have not previously been reported in the literature.

One mechanism by which PIM-1 regulates the cell cycle is through PIM-1 phosphorylation of Cdc25A and Cdc25C, which results in an increase in cells in G1/S and G2/M transition [22, 23]. In addition, PIM-1 can phosphorylate P21^cip1/waf1^ and P27^kip1^, which are involved in regulation of the cell cycle [24, 25]. PIM-1 is also involved in regulating apoptosis through blockade of multiple apoptotic signals. PIM-1 phosphorylates Bad at Ser112, which results in proteasomal degradation, thus protecting cells from the pro-apoptotic effects of Bad [26]. PIM-1 can also regulate apoptosis by phosphorylating ASK1 at Ser83 [27] or phosphorylating PRAS40 at Thr246 [28]. PIM-1 likewise plays a role in the regulation of chemoresistance. PIM-1 increases the phosphorylation of ABCG2 at Thr362, and downregulation of PIM-1 increases the chemosensitivity of prostate cancer cells [29]. PIM-1 has also been shown to regulate P-glycoprotein (Pgp, ABCB1) by protecting Pgp, a 150 kD protein, from degradation and enabling Pgp glycosylation and cell surface translocation [30]. These studies may partly account for the effects of PIM-1 on pancreatic cancer but do not explain the mechanisms by which PIM-1 regulates the sensitivity of cells to erlotinib or affects the expression of pancreatic cancer stem cell markers.

Cancer cells present different mechanisms of drug resistance including innate mechanisms that result in modulation of intracellular signaling pathways [31]. One potential mechanism may be that PIM-1 and the EGFR signalling pathway form a feedback loop. Siu and colleagues found that knockdown of PIM-1 upregulated the expression of MIG6, a negative regulator of EGFR signalling, which then inactivated EGFR signalling [20]. The EGFR signalling pathway also plays a role in the regulation of PIM-1 expression. Stimulation of the EGFR pathway with EGF or TGF-α induced PIM-1 upregulation and nuclear translocation in head and neck squamous cell carcinoma (HNSCC) cell lines [32]. Conversely, downregulation of EGFR in HeLa cells may suppress PIM-1 mRNA expression [33]. Our study is the first to verify the feedback loop between PIM-1 and the EGFR signalling pathway in pancreatic cancer. Activation of the EGFR signalling pathway resulted in cell proliferation, anti-apoptosis, angiogenesis, metastasis, and chemoresistance to gemcitabine and EGFR-TKIs and promoted the activity of stem cells in various cancers [34, 35]. Thus, the potential mechanism by which PIM-1 affects sensitivity to the chemotherapy drug erlotinib or the expression of pancreatic cancer stem cell markers in pancreatic cancer may be by regulating the EGFR signalling pathway. Numerous PIM‑1 inhibitors, such as flavonoid inhibitors, ETP‑45299, SGI‑1776 and AZD1208, have been developed [36–39]. SGI‑1776, as a first-generation inhibitor, has high anti-tumour activity in vivo and in vitro by inhibiting FLT3, cyclin D1, MCL, Myc and Pgp [40, 41]. Thus, the combination of PIM-1 inhibitor with erlotinib may be new method for pancreatic cancer therapy in future investigations [26].

PIM-1 levels significantly increased in not only cancer tissues but also cancer stroma as reported in a previous study [11]. The roles and mechanisms of increased levels of PIM-1 in stroma have not been elucidated in pancreatic cancer. Zemskova et al. found that PIM‑1 was highly expressed in human prostate cancer stroma [42]. PIM-1 could induce fibroblast cells to secrete extracellular matrix, collagen 1A1, chemokine CCL5, and platelet‑derived growth factor receptor to enhance the ability of fibroblasts to differentiate into myofibroblasts and express known markers of cancer-associated fibroblasts (CAFs) [42]. Additionally, PIM-1 promoted prostate cancer cell migration and invasion by phosphorylating CXCR4 at Ser‑339 [43]. These findings suggest that PIM‑1 may have a significant potential in cancer metastasis by crosstalk with the tumour microenvironment. The roles and mechanisms by which PIM-1 levels increase in pancreatic cancer stroma should be demonstrated in future studies.

The prognostic value of PIM-1 levels in cancer tissues remains controversial. Peng et al. demonstrated that the PIM-1 expression level in colon cancer tissues was not prognostic [44]. Liu et al. found that high PIM-1 expression levels were associated with poor prognosis in patients with oesophageal squamous cell carcinoma [45]. However, Reiser-Erkan and colleagues showed that the presence of PIM-1 in pancreatic cancer cells had a favourable prognostic impact [11]. In the present study, we demonstrated that the PIM-1 expression level in tissue was an independent poor prognostic factor, which is consistent with the oncogenic role of PIM-1 in pancreatic cancer. Further studies are needed to investigate the prognostic value of PIM-1 in cancers.

Then, we analysed expression levels and clinical value of plasma PIM-1 for the first time. We found that plasma PIM-1 levels in patients with pancreatic cancer were significantly increased and were better than CA19-9 levels in differentiating patients with pancreatic cancer from healthy volunteers and patients with chronic pancreatitis; thus, the plasma PIM-1 level may serve as a new circulating marker for the diagnosis of pancreatic cancer. Furthermore, we found that plasma PIM-1 levels were associated with TNM staging. Patients with high plasma PIM-1 levels had advanced-stage tumours. Therefore, the plasma PIM-1 level could be a new marker for TNM staging of pancreatic cancer. We also found that a high plasma PIM-1 level was an independent adverse prognostic factor and could serve as a new prognostic marker for pancreatic cancer.

The present study has several limitations. First, the pancreatic cancer tissues analysed in the study were obtained from surgical resection, so there was inherent bias to analyse the relativity between PIM-1 expression levels and TNM stages because few patients were eligible for surgical resection. Second, the pancreatic cancer tissue and plasma samples available in our study were not paired. Further studies are needed to investigate the correlation of PIM-1 expression levels between tumour tissue and plasma. Third, we did not measure EGFR expression levels in pancreatic cancer tissues or plasma. The feedback loop of PIM-1 and the EGFR signalling pathway would be strengthened if the relevance of expression levels between PIM-1 and EGFR could be verified in pancreatic cancer.

In conclusion, PIM-1 and the EGFR pathway form a feedback loop, which contributes to the malignancy of pancreatic cancer. PIM-1 levels in tissues and plasma were independent prognostic factors, and PIM-1 may be a new prognostic biomarker for pancreatic cancer. Plasma PIM-1 levels also displayed potential diagnostic value in pancreatic cancer.

## Conclusions

PIM-1 is upregulated in pancreatic cancer tissues and plasma and may serve as an independent adverse prognostic factor for pancreatic cancer. Knockdown of PIM-1 expression in pancreatic cancer cells suppressed proliferation, induced cell cycle arrest, enhanced apoptosis, resensitized cells to gemcitabine and erlotinib treatment, and inhibited ABCG2 and EZH2 mRNA expression. Thus, PIM-1 may be a biomarker and potential therapeutic target in pancreatic cancer.

## Additional files

Additional file 1: Figure S1. The effect of PIM-1 siRNA on PIM-1 expression. PDAC cells were transfected with PIM-1 siRNA or control for 48 h. Total proteins were harvested, and PIM-1 protein expression levels were detected by western blot. (TIF 123 kb) Additional file 2: Table S1. Correlations of PIM-1 levels in tissues and clinicopathological parameters. (XLSX 11 kb) Additional file 3: Table S2. Univariate and multivariable analyses of factors predictive of poor overall survival in patients with pancreatic cancer (tissues). (XLSX 12 kb) Additional file 4: Table S3. Correlations of plasma PIM-1 levels and clinicopathological parameters. (XLSX 11 kb) Additional file 5: Table S4. Univariate and multivariable analyses of factors predictive of poor overall survival in patients with pancreatic cancer (plasma). (XLSX 12 kb)

## Abbreviations

ABCG2: Adenosine triphosphate-binding cassette superfamily G member 2
EGFR: Epidermal growth factor receptor tyrosine kinase
EZH2: Enhancer of zeste homologue 2
PDAC: Pancreatic ductal adenocarcinoma
PNET: Pancreatic neuroendocrine tumour
